# Platelet-Rich Plasma Treatment for Chronic Respiratory Disease

**DOI:** 10.7759/cureus.33265

**Published:** 2023-01-02

**Authors:** Aaron D Knight, Sandeep Kacker

**Affiliations:** 1 Pharmacology and Therapeutics, Medical University of the Americas, Devens, USA

**Keywords:** anti-inflammation, tissue regeneration, covid-19 and pulmonary fibrosis, copd (chronic obstructive pulmonary disease), chronic respiratory disease, platelet-rich plasma (prp)

## Abstract

This study was conducted to test the hypothesis that platelet-rich plasma (PRP) therapy in chronic respiratory disease patients will cause lung regeneration, thereby slowing the progression of the disease.

We performed a search to obtain pertinent articles on the following electronic databases: Google Scholar, PubMed, NCBI, Medscape, and clinicaltrials.gov. Keywords used during in search included “Platelet Rich Plasma” AND “Chronic Respiratory Disease” AND/OR “Chronic Obstructive Pulmonary Disease”. A total of 15 articles were chosen for this paper, published from 2011 to 2021, and included case series, lab studies, animal studies, cohort studies, and clinical trials. All statistical data were considered significant if the p-value was less than 5%, or 0.05.

Our findings confirmed that PRP therapy successfully caused anti-inflammatory effects and acceleration of tissue regeneration, resulting in improved lung function. This, in turn, slowed the progression of the disease and led to an improved quality of life.

Not all chronic respiratory disease patients present in the same manner, but the connecting link is the damaged tissue of the lungs, causing issues with the functionality of the lungs. By adjunctively treating patients with PRP, the high concentration of platelets and their secreted growth factors can help induce an acceleration of healing and regeneration of pulmonary tissue. This, in turn, can slow the progression of the disease, which could lower the overall mortality rate in chronic respiratory disease patients. More studies should be conducted on this topic, specifically large, double-blinded, randomized human trials with controls, to further assess the efficacy and beneficial effects of PRP treatment on the lungs.

## Introduction and background

It is estimated that 37 million Americans live with a chronic respiratory disease, and this dreadful condition led to the deaths of over 152,000 people in the United States in 2020 [1,2]. Chronic respiratory disease encompasses many disorders, including chronic obstructive pulmonary disease (COPD), asthma, interstitial lung fibrosis, and others [3]. Historically, of all the respiratory disorders, COPD is by far the deadliest. In 2020, 5% of Americans had a diagnosis of COPD, emphysema, or chronic bronchitis, which caused over 140,000 deaths [3]. COPD is the third leading cause of death worldwide, killing more than 3.23 million people in 2019 [4]. COPD is a common lung disorder that causes many abnormalities of the airflow in and out of the lungs. COPD comprises two main disorders: emphysema and chronic bronchitis [5]. Emphysema results from the destruction of alveoli, which are the terminal points of the lung allowing for the proper gaseous exchange of oxygen and carbon dioxide [5]. Chronic bronchitis is caused by excess mucus production in the lungs, leading to mucus plugging within the airways [5]. Both disorders are usually associated with smoking or exposure to tobacco, but they can also result from occupational exposures, restrictions of normal lung growth during childhood or adolescent years, and even genetic causes [5]. While both disorders are different, they are linked by the common symptoms of shortness of breath and difficulty breathing. Diagnosing COPD can be challenging; the most effective diagnostic test involves measuring lung function through spirometry [6]. The most commonly used pulmonary function tests together with spirometry for diagnosing COPD are the FEV1 (forced expiratory ventilation in the first second of expiration), FVC (forced vital capacity in an effortful expiration), and FEV1%/FVC (the proportion of FEV1 in an entire effortful expiration) [6]. Typically, FEV1%/FVC should be greater than 70%, and a value less than 70% would be indicative of COPD [6].

While there are many medications and treatments for COPD, there is no definitive cure for the disease. Treatment modalities like oxygen therapy and medications such as bronchodilators, anticholinergic agents, and inhaled/oral steroids are aimed at achieving symptomatic relief and preventing or treating disease exacerbations or flare-ups [7]. However, currently, there is no cure for the disease, and progression to mortality is a major concern.

While COPD is the most common lung disorder historically, with the emergence of the ongoing coronavirus disease 2019 (COVID-19) pandemic, caused by severe acute respiratory syndrome coronavirus 2 (SARS-CoV-2), many other variants of lung disorders have been reported. To date, there have been over 95 million confirmed cases in the US, killing over one million Americans [8]. The most common severe complication of COVID-19 is acute respiratory distress syndrome (ARDS) [9]. Though this pandemic is still ongoing, the long-lasting effects on recovered COVID-19 patients are already starting to be seen. ARDS could lead to many chronic effects, including lung fibrosis and scarring [10]. ARDS is caused by the buildup of fluid within the alveoli of the lungs, which can cause acute lung injury [10]. While pulmonary fibrosis affects the interstitium of the lung (cells that surround structures inside the lung) and COPD affects the alveoli and actual airways, they both result from lung damage and are considered terminal illnesses [11,12].

Chronic respiratory disease progression is unfortunately inevitable. While the current protocol of medications focuses on relieving symptoms and preventing and controlling exacerbations, many researchers are hopefully looking to mesenchymal stem cell (MSC) therapy for lung regeneration [13]. The current ways to obtain MSC are either via umbilical cords/embryonic tissue, bone marrow, or adipose tissue [13,14]. While MSC therapy looks promising, there are many challenges in obtaining these cells. Collecting these cells from umbilical cords or embryonic tissue is controversial in nature and acquiring MSC from bone marrow or adipose tissue requires a painful, invasive procedure with many possible complications [14]. An alternative is platelet-rich plasma (PRP). PRP is essentially plasma and serum, without erythrocytes, or red blood cells (RBC) [15]. To obtain PRP, a standard blood draw is done, and the blood is centrifuged to separate the RBCs from the remaining plasma [15]. This remaining plasma has a higher concentration of platelets, which contain many growth factors to aid in tissue repair. Some of these factors include platelet-derived growth factor (PDGF), insulin-like growth factor (IGF), vascular endothelial growth factor (VEGF), epidermal growth factor (EGF), transforming growth factor (TGF), and platelet-derived angiogenesis factor (PDAF); all of which play key roles in tissue regeneration by accelerating the healing process [15]. PRP’s application in orthopedic injuries, oral and maxilla-facial surgeries, and dermatologic and cosmetic surgeries to accelerate healing has been shown to be beneficial [16]. While the exact mechanism of how PRP regenerates tissue is yet to be understood, the effects that platelets have on pulmonary endothelium by releasing these growth factors include maintenance of barrier integrity via cell proliferation, enhancement of endothelial cell growth, and the neutralization of barrier permeability [17].

The purpose of this study is to determine if patients with COPD or other chronic lung disorders can benefit from PRP therapy, which accelerates lung regeneration. Although there is vast evidence of clinical progression in these patients, the current treatment of COPD is symptomatic in nature, and most patients deteriorate to the point where they either require lung transplantation or unfortunately die [7]. Since PRP therapy is quite effective in many fields of medicine in terms of accelerating natural tissue healing, the benefits of its application for chronic respiratory disease patients will be thoroughly reviewed in this study [16,18].

Methods

The following databases were used to obtain relevant material for this study: Google Scholar, PubMed, NCBI, Medscape, and clinicaltrials.gov. The specific keywords used to search for relevant articles were “Platelet Rich Plasma” AND “Chronic Respiratory Disease” AND/OR “Chronic Obstructive Pulmonary Disease”. To narrow down the material to a level of absolute relevance, the additional keywords used were “Autologous PRP” AND “Lung Regeneration”, but NOT “Stem Cell”.

Studies that were considered relevant included those that were published in or after 2006. Different types of studies were chosen, including case series, controlled clinical trials, non-controlled clinical trials, cohort studies, animal studies, and lab studies. Initially, the choice of studies was limited to human trials, but since PRP therapy is a rather new field of medicine and there is a limited number of published articles available regarding lung regeneration via PRP, case series, non-controlled clinical trials, animal trials, and lab studies were also considered relevant for this review.

Articles were excluded if they involved terms relating to “mesenchymal stem cells”, “drug therapy”, and “musculoskeletal injuries”. However, one study regarding “musculoskeletal injuries” was included as it helped explain the anti-inflammatory and tissue-regenerating capabilities of PRP therapy. Articles were excluded if proper statistics were not available. The exclusion criteria also included any articles that provided access to study abstracts only and reports from before 2006.

Initially, 32 articles were chosen for review. However, after eliminating duplicates and analyzing bias and the quality of the studies, this was reduced to 15 relevant articles, which were used to explore the topic and establish the hypothesis of this study. The reason for choosing a high number of primary articles was due to the limited availability of studies with a high level of evidence. Thus, including many reports with a lower level of evidence assisted in preventing biases and preferred outcomes.

All statistical data presented in the results section of this paper were considered significant if the p-value (the value that a given outcome could have occurred by chance alone) was less than 5%, or 0.05. Statistical analysis of the results section was performed using the following tests: t-test, analysis of variance (ANOVA), chi-squared test, two-tailed Wilcoxon signed-rank test, Shapiro-Wilk test, Fisher's exact test, and analysis of logistic regression. All data were considered statistically significant unless otherwise stated. Statistics and other data in the introduction were sourced from the World Health Organization (WHO), the Centers for Disease Control and Prevention (CDC), the American Lung Association (ALA), the Cleveland Clinic, the Mayo Clinic, and John Hopkins Coronavirus Resource Center to better outline the need for further research in the treatment of PRP in chronic respiratory disease patients. All primary articles used in this study are presented in a table in the next section, which provides a summary of the following: authors of the study, publication date, study design, the study population, therapy/exposure used in the study, the outcomes of the study, and the level of evidence. All published material was included in this table, except for articles that were used for background information provided in the introduction section.

## Review

Results

As previously mentioned in the introduction, the goal of this paper is to evaluate if patients with COPD or other chronic lung disorders can benefit from PRP therapy, which accelerates lung regeneration and slows the progression of the disease. If the natural progression of the disease is slowed, the belief is this will decrease mortality. However, just like stem cell therapy, PRP is not a cure for the disease, but rather potentially an adjunctive therapy to better control the mortality rate. Our review involved reviewing 15 articles that explored the use of PRP in chronic respiratory disease patients.

The information we present here explores the results of the 15 researched articles on PRP therapy in chronic respiratory disease patients. Four of the reviewed articles discuss the association between PRP use and the improvement of lung function and decreased exacerbations of the disease. The other 11 articles delve into the relationship between PRP therapy and tissue regeneration and anti-inflammatory effects. The remaining part of this section summarizes the results and data from the 15 articles selected and reviewed.

From 2017 to 2020, Rubio conducted a prospective cohort study to establish a possible relationship between PRP use and the improvement of lung function and decreasing symptoms [19]. A total of 419 COPD patients provided a baseline FEV1/FVC using spirometry, as well as a self-reported questionnaire on quality of life [19]. The questionnaire used was the Clinical COPD Questionnaire (CCQ), which is validated and endorsed by the Global Initiative for Chronic Obstructive Lung Committee (GOLD) [19]. The CCQ is a 10-question survey, with a score for each question ranging from 0 to 6, where 0 indicates “never”, while 6 indicates “always” [19]. The higher the numerical value of each component, the worse it is for the patient [19]. The CCQ quantifies three important aspects: COPD/respiratory symptoms, mental health status, and functional health state [19]. These patients all had their blood drawn and centrifuged to formulate PRP and were then infused back into them intravenously [19]. All 419 patients completed a CCQ survey at three, six, and 12 months post-PRP treatment [19]. Regarding COPD symptoms, the average CCQ scores decreased from 3.56 at baseline to 2.80 at three months, 2.60 at six months, and 2.78 at 12 months post-PRP [19]. For mental health, the average CCQ score also decreased from 3.83 at baseline, to 2.65, 2.26, and 2.48, at three, six, and 12 months post-treatment, respectively [19]. Functional health scores also decreased from 3.47 at baseline, to 2.87, 2.88, and 0.62, at three, six, and 12 months post-treatment, respectively [19]. A paired sample t-test conducted on each pair of baseline and follow-up scores showed significantly decreased numbers with a p-value of less than 0.001, making this statistically significant [19]. Although the baseline FEV1 was ascertained for all 419 patients, only 150 of these patients fulfilled a three-month FEV1/FVC check [19]. For these 150 patients, the FEV1 increased from a baseline of 32.65% to 34.3% at three months post-PRP, with an average increase of 7.83% [19]. Of note, 101 out of the 150 patients who retested their FEV1 at three months had either improved scores or no change [19]. A paired sample t-test was conducted to assess the difference between baseline and three-month FEV1/FVC, which was also statistically significant with a p-value of 0.003 [19].

Another cohort study performed by Rubio assessed the changes in FEV1/FVC in 281 COPD patients after autologous PRP was administered intravenously [20]. A baseline FEV1//FVC was measured before any treatment. The 281 patients were classified into two groups: Group A had 150 patients and their FEV1% was reevaluated after three months, while Group B had 131 patients, whose FEV1% was remeasured after 12 months [20]. The PRP infusion was prepared in the same way as in her previous study, mentioned above. Group A had an increase of FEV1/FVC from 33.2% at baseline to 34.7% at three months, with an average increase of 7.2% per patient [20]. Group B also showed an increase in their FEV1/FVC from 35.8% at baseline to 38.1% at 12 months, with an average rise of 6.88% per patient [20]. Using the two-tailed Wilcoxon signed-rank test and the Shapiro-Wilk test, results were shown to be statistically significant (p-value less than 0.05) [20]. Analysis of variance (ANOVA) was conducted for both groups and the results showed the following: Group A had F=1.149 and a p-value of 0.011, while Group B had F=1.130 and a p-value of 0.004 [20]. Together both groups showed statistically significant increases in their respective group’s FEV1%, signaling an improved lung function after the PRP therapy [20].

A case series conducted by Coleman and Rubio in 2015 measured the changes in COPD patients and their quality of life after autologous PRP was administered intravenously [21]. This case series involved 568 patients with COPD stages 2-4 [21]. Each participant had a CCQ assessed at baseline to quantify their quality of life and again at six months post-therapy [21]. This study classified patients into different groups. Group 1 (n=495) had a single veinous harvest of PRP and was administered the autologous PRP for three consecutive days [21]. Group 2 (n=17) had a double veinous harvest; the first PRP harvest was administered for three consecutive days; then three months later, the second harvest of PRP was done and administered for another three consecutive days [21]. Group 3 (n=36) had a single veinous harvest and reinfusion for one day, a bone marrow harvest and reinfusion on the second day, and a reassessment of the bone marrow wound on the third consecutive day [21]. Group 4 (n=30) was the “booster” group, which followed the same protocols as group 1; however, with disease progression, a second PRP infusion was conducted [21]. It is important to note that higher scores indicate a poorer quality of life, and lower scores show a better quality of life for the patient. Group 1 had a decrease in their COPD symptoms from 3.5 at baseline to 2.2 at six months, a decrease in mental health scores from 3.8 at baseline to 2.3 at six months, and a decrease in functional health scores from 3.7 at baseline to 2.8 at six months [21]. Group 2 had a decrease in their COPD symptoms from 2.9 at baseline to 1.4 at six months, a decrease in mental health scores from 4.2 at baseline to 1.9 at six months, and a decrease in functional health scores from 2.7 at baseline to 1.9 at six months [21]. Group 3 had a decrease in their COPD symptoms from 3.0 at baseline to 1.9 at six months, a decrease in mental health scores from 3.6 at baseline to 2.0 at six months, and a decrease in functional health scores from 3.7 at baseline to 2.7 at six months [21]. Group 4 had a decrease in their COPD symptoms from 2.9 at baseline to 2.4 at six months, a decrease in mental health scores from 3.3 at baseline to 1.8 at six months, and a decrease in functional health scores from 3.2 at baseline to 2.5 at six months [21]. The results from the CCQ were deemed clinically significant if there was a difference of 0.4 or more at the follow-up [21]. Utilizing the ANOVA, with a p-value of 0.05, together with a significant change in the CCQ at the six-month follow-up, this case series showed clinically and statistically significant changes in the quality of life of these COPD patients [21]. Although there were differences based on the group treatment type, the PRP infusion treatment positively affected each component of the CCQ at six months compared to the baseline [21].

In a case series by Karina et al., activated platelet-rich plasma (aPRP) was administered to seven COVID-19 patients suffering from ARDS [22]. The aPRP was created using patients’ blood that was centrifuged, and the remaining plasma was combined with a calcium activator [22]. Before administration, blood clots were removed and the remaining solution was reinfused into patients on days one, three, and five while in the intensive care unit [22]. Before the administration of the aPRP, a baseline lab level of interleukin-1B (IL-1B) was obtained, together with their PaO_2_/FiO_2_ and lung injury score [22]. IL-1B is a useful biologic marker that can help determine the inflammation in the body [22]. PaO2/FiO_2 _measures the ratio of arterial oxygen pressure dissolved in the blood compared to the level of fractional inspired oxygen, and a completely normal level is between 500-600 mmHg, with a value under 300 mmHg being diagnostic of ARDS [22]. The seven patients were subdivided into two groups to distinguish between the different IL-1B levels: three had severe disease and four were critical [22]. On days two, four, and six (after each aPRP treatment), these levels were each remeasured. Three out of the four critical patients ended up dying shortly after day six [22]. The three severe patients’ IL-1B levels decreased on average from 4.71pg/ml to 2.48pg/ml from day one to six, while the four critical patients’ IL-1B levels increased on average from 3.095pg/ml to 18.77pg/ml within the same timeframe [22]. Although 75% of the critical patients died, this development was not statistically significant based on a paired t-test (p-value was greater than 0.05) [22]. While evaluating the lung injury scores and the change in PaO_2_/FiO_2_, all seven patients were grouped together. The lung injury scores increased from 5.33 at baseline to 6.0 on day 6 [22]. While there was a net increase in the scores, after the second aPRP infusion, they decreased from 6.50 on day four to 6.0 on day six [22]. However, these changes were also not statistically significant as per a paired t-test (p-value was above 0.05) [22]. The final measurement conducted - the difference in PaO_2_/FiO_2_ - did show meaningful results [22]. The seven COVID-19 patients had an average baseline PaO_2_/FiO_2_ of 71.33 mmHg, which was diagnostic of ARDS [22]. On day six, however, these scores increased to 144.97 mmHg on average [22]. This value was statistically significant based on a paired t-test, with a p-value less than 0.05 [22]. While these scores never went beyond 300 mmHg, signaling that they were still experiencing ARDS, their ARDS was less severe, highlighting improved lung function [22].

From 2018 to 2020, Pires et al. conducted a randomized controlled animal trial involving 37 racehorses suffering from exercise-induced pulmonary hemorrhage to evaluate PRP treatment efficacy [23]. Most racehorses were evaluated post-race, which included endoscopy, as it is quite common for these horses’ airways to become inflamed and edematous [23]. Thirty-seven horses that showed signs of mucosal edema, airway inflammation, and visible airway bleeds were included and were randomly assigned to either placebo (n=14) or treatment group (n=23) [23]. Three days after the races, blood was collected for PRP and bronchoalveolar lavage (BAL) was conducted, with specimens to be used as control. The placebo group was administered 10 mL of 0.9% normal saline, while the study group was given 10 mL of autologous PRP; both groups were given 10 mL intrabronchially [23]. Five weeks after the intrabronchial administration, all the horses participated in another race and were again evaluated endoscopically post-race with BAL. The BAL specimens were all scored from 0 to 5, with 0 indicating no bleeds or inflammation and five being the worst result [23]. The 14 placebo horses had a mean increase from 2.0 after the first race to 2.5 after the second race, while the PRP-treated horses had a mean decrease from 3.21 to 1.52, respectively [23]. This was statistically significant after analysis revealed p=0.002 when comparing the pre-and post-race results of the PRP group to those of the placebo group [23].

In 2015, Mammoto et al. conducted a controlled animal trial to assess whether PRP could be used to promote the angiogenesis of the lung [24]. Since it has been recognized that PRP promotes endothelial cell sprouting in vitro and in vivo and that endothelial cells stimulate alveolar morphogenesis, this animal study assessed whether PRP can cause better lung remodeling [24]. The study involved six mice that had undergone surgical left pneumonectomy and PRP was used to promote overall better compensatory lung growth in the remaining right lungs. Six other mice were used as controls and, after the unilateral pneumonectomy, the following parameters were compared with the control group: right lung weight, static lung compliance, pulmonary vascular permeability, alveolar-arterial oxygen difference, and exercise capacity [24]. After seven days, some mice underwent microscopy of the lung as well. Once the surgery was complete, PRP extract was given to the mice every day for 14 days, via intraperitoneal injection, and all measurements were reassessed at seven and 14 days post-surgery [24]. As expected, in all mice that had undergone unilateral left pneumonectomy, the right cardiac lung lobe increased significantly by 1.4-fold from days seven to 14 post-surgery (measured via the ratio of right cardiac lung lobe weight to total mouse weight) [24]. However, in the PRP-treated mice post-surgery, their ratio significantly increased by 14% compared to the control group (pneumonectomy without PRP treatment) [24]. Regarding right lung compliance, in PRP-treated mice, the total right lung compliance was 30% greater than the control group post-surgery [24]. Lung vascular permeability was measured by intravenously injecting dye and measuring the leakage into the alveolar space. However, there was no change between the PRP-treated group and the control group in terms of permeability, indicating that PRP did not cause inflammation of the tissue [24]. There was also no difference between the study and control group with regard to alveolar-arterial oxygen. However, microscopy using hematoxylin and eosin (H&E) stain showed thickened alveola septa, a decrease in the size of alveolar space, and an increase in the number of alveoli in PRP-treated mice post-pneumonectomy compared to the control group [24]. Additionally, the blood vessel density of the lung was analyzed using CD31, a blood vessel biomarker, and showed increased blood vessel density in the PRP group compared to controls [24]. These results indicate that PRP accelerates vascular and alveolar regeneration and remodeling post-pneumonectomy [24]. All analyses were performed by masked observers, to ensure unbiased findings. A student’s t-test and ANOVA were conducted, which showed the analysis of all measurements to be statistically significant (p-value was less than 0.05) [24].

Salama et al. conducted a randomized controlled trial from 2015 to 2017 in order to assess the efficacy of nebulized autologous PRP in smoke inhalation patients [25]. The study included a total of 40: 20 in the study group and 20 acted as the control group [25]. Each patient had between 25-50% of total body surface area burns, plus smoke inhalation lung injuries [25]. Each group, regardless of PRP, received the standard treatment regimen. Each patient required mechanical ventilation via intubation within 48 hours of admission. The study group was given autologous PRP via nebulization as an adjunctive treatment [25]. Throughout each patient’s hospital stay, the following variables were recorded for statistical use: day of extubation (patient no longer requiring mechanical ventilation), mortality rate, hospital length of stay, and PaO_2_/FiO_2_ [25]. The mean intubation length was seven days for the nebulized PRP group, compared to 14 days in the control group [25]. The average hospital stay was also less in the PRP group (15 days), compared to controls (23 days) [25]. The mortality rate was 10% in the PRP group and 20% in the control group [25]. The average PaO_2_/FiO_2_ was 36.24 in the PRP group and 25.17 in the control group [25]. Additionally, in the nebulized PRP group, there was a significant difference in biological changes in the upper airway, compared to the control group, which manifested as follows: decreased edema, decreased mucus formation, and decreased tissue inflammation [25]. The following statistical tests were used to ensure the value of this cohort study: Pearson's correlational coefficient for analyzing the association between different parameters, student’s t-test to assess the differences between the two groups, and logistic regression for evaluating all combined factors (age, gender, total body surface areas of burns, and PaO_2_/Fio_2_ ratio) [25]. The p-value level of significance was less than 0.01 for all tests, showing a statistically significant difference [25].

A controlled animal study performed by Gómez-Caro et al. assessed the relationship between PRP use and airway healing as well as the decrease in possible anastomotic complications [26]. This study involved 15 adult Yorkshire Duroc pigs. These pigs were randomly divided into three groups, with five pigs in each group. Group 1 was the sham treatment group; each pig underwent cervicotomy and cervical and tracheal dissection without resection [26]. This group acted as the control group. Group 2 underwent cervicotomy and cervical dissection with resection of 50% of the tracheal total length with end-to-end anastomosis [26]. This group was the non-PRP group. Group 3 underwent group 2’s protocols with the addition of PRP applied over the surgical area, including the anastomoses [26]. Blood was extracted from the animals via a jugular vein central line and was centrifuged to create the separation of RBCs and plasma [26]. The RBCs were removed and the PRP was left. A small sample of the PRP was analyzed and the remaining PRP was used during the surgery. This central line was also the point to extract blood for assessment of growth factors at one and six hours post-surgery, and laser Doppler flowmetry was used to measure the blood flow rates during the surgery and 30 days post-surgery [26]. After 30 days, the animals’ tracheas were viewed under microscopy and blindly assessed, to ensure unbiased findings [26]. All surgeries were completed successfully without complications; however, one pig in Group 2 (non-PRP) died at nine days due to pneumonia [26]. The sampled PRP showed a platelet count of 638 x 10^9^ per cubic millimeter of blood (mm^3^), while the whole blood sample showed 176 x 10^9^ mm^3^ (p=0.02) [26]. The following platelet-derived growth factors were assessed at one and six hours post-surgery: total growth factor-β (TGF-β), EGF, and VEGF [26]. Group 1 (sham) had an increase in TGF-β from 0.58 ng/ml at one hour to 0.69 ng/ml at six hours, an increase in EGF from 0.21 ng/ml at one hour to 0.23 ng/ml at six hours, while VEGF stayed the same (0.10 ng/ml) at one and six hours [26]. Group 2 (non-PRP) had an increase in TGF-β from 0.63 ng/ml at one hour to 0.66 ng/ml at six hours, a decrease in EGF from 0.50 ng/ml at one hour to 0.23 ng/ml at six hours, and an increase in VEGF from 0.12 ng/ml at one hour to 0.14 ng/ml at six hours [26]. Group 3 (PRP) had the same TGF-β values (0.86 ng/ml) at one and six hours, a decrease in EGF from 0.86 ng/ml at one hour to 0.69 ng/ml at six hours, and a decrease in VEGF from 0.30 ng/ml at one hour to 0.18 ng/ml at six hours [26]. The PRP group consistently showed the highest values with regard to platelet-derived growth factors (p=0.03), indicating a possible acceleration of healing [26]. Laser Doppler flowmetry showed that the average trans-anastomotic blood flow rate in Group 2 was +3.8%, while it measured +15.6% in Group 3 (p=0.04) [26]. After 30 days, the tracheas were blindly assessed by pathologists and showed higher vessel density and epithelial thickness in the PRP group, compared to the non-PRP group; but this was not statistically significant (p=0.08) [26]. All data values were determined to be significant via ANOVA and student’s t-test, except for the pathology report [26].

Maher et al. did a controlled animal study to assess the efficacy of PRP usage for the treatment of amiodarone-induced pulmonary fibrosis [27]. Amiodarone, a very useful medication in cardiology, is known to have many adverse effects, including pulmonary fibrosis [27]. This trial used 70 adult albino rats in total, divided into three groups, with each group consisting of 20 rats, while 10 rats functioned as PRP donors [27]. Group 1 acted as the control group. Group 2 (non-PRP) was given amiodarone via intraperitoneal injection daily for three weeks, followed by phosphate-buffered saline (PBS) twice weekly for three weeks via intra-peritoneum [27]. Group 3 (PRP) had a similar protocol to Group 2: amiodarone daily for three weeks intraperitoneally, followed by PRP twice weekly for three weeks [27]. The following data were collected between weeks four and six: hematological analysis, BAL fluid collection, and antioxidant biomarkers [27]. Glutathione reductase and malondialdehyde (MDA) were used as the antioxidant markers. Glutathione reductase is an important enzyme used to treat oxidative stress, and MDA is an important marker signifying if oxidative stress has occurred [27]. At the end of the study, lung tissue was examined using microscopy. All statistical analyses were performed using ANOVA. The study showed that Group 2 (non-PRP) experienced a significant decrease in white blood cells (WBC) and RBC compared to the control group [27]. However, Group 3 showed a significant increase in WBC at weeks four to six, and an increase in RBC at weeks five to six when compared to Group 2 [27]. Glutathione reductase levels were significantly decreased in Group 2 at weeks four to six, compared to the control group [27]. However, in Group 3, there was a significant increase in glutathione reductase at weeks five to six when compared to Group 2 [27]. Both Groups 2 and 3 showed significantly increased levels of MDA in the fourth week when compared to the control group [27]. However, in Group 2, this level remained high in weeks five to six, while Group 3’s MDA levels significantly decreased in weeks five to six, when compared to each other [27]. The BAL fluid was collected and stained with Giemsa stain and was examined under the microscope, as was the lung tissue. Microscopy determined that Group 2 (non-PRP) had significant leukocytosis, abundant macrophages, and suffered from interstitial pneumonia with emphysematous areas and bronchiolitis [27]. Group 3 (PRP) had a significantly lower leukocytosis with normal lung tissue, which was evident in PRP’s acceleration of healing [27]. All data were proven to be statistically significant via ANOVA with p-values less than 0.05 [27].

In another animal trial conducted by Dzyekanski et al., 10 racehorses with recurrent cough, wheezing, and other pulmonary symptoms were administered intrabronchial PRP for treatment analysis [28]. The BAL fluid was collected before treatment and again at seven days post-treatment. After analyzing the BAL specimen, the horses were grouped based on their diagnosis: recurrent airway obstruction (RAO) (n=2), inflammatory airway disease (IAD) (n=5), and normal lung (NL) (n=3) [28]. Although the three horses in the “normal” group experienced pulmonary symptoms, their BAL sample was inconclusive for diagnosis [28]. The PRP was prepared similarly to the previous studies reviewed and was administered into the right and left main bronchus (intrabronchial), using endoscopic guidance [28]. The BAL fluid was examined and analyzed for tracheal mucus grade and relative neutrophil count [28]. After examining the BAL fluid on day seven, the results were determined. The IAD group had an average tracheal mucus reduction of 2.4, compared to a reduction of 1.4 in the RAO group (p=0.034) [28]. Additionally, the relative neutrophil count was significantly decreased in the IAD group by 13.0, compared to 5.0 in the RAO group (p=0.014) [28]. The IAD horses were the only group to show clinically significant improvement in pulmonary effects in a physical exam, while both the RAO and NL groups showed little to no clinical improvement [28]. The NL group also showed very little change in mucus grade reduction and relative neutrophil reduction, per their BAL sample [28]. This trial showed the anti-inflammatory effect and improvement of PRP in IAD racehorses [28]. All statistical data were analyzed by a paired student’s t-test, to identify statistical significance [28].

In a human clinical trial conducted by Karina et al., the researchers investigated the safety and efficacy of aPRP as an adjunctive treatment in severely ill COVID-19 patients [29]. Ten COVID-19 patients suffering from ARDS were enrolled in the study and were given aPRP on days one, three, and five, via intravenous infusion [29]. The PRP was prepared similarly as in previously reviewed studies; however, a calcium activator was added to the mixture for activation [29]. On days two, four, and six (post-PRP), the following lab values were ascertained: C-reactive protein (CRP), neutrophil count, lymphocyte count, lymphocyte-to-CRP ratio (LCR), and neutrophil-to-lymphocyte ratio (NLR) [29]. A baseline level of these labs was also collected before any aPRP therapy, which was used to identify if aPRP had any anti-inflammatory effects in severely ill COVID-19 patients [29]. All statistical lab value changes were analyzed with a paired t-test, and the Shapiro-Wilk test was used for data distribution [29]. The average CRP level on day one was 10.89 and decreased to 2.53 on day six (p=0.005); the mean neutrophil count was 81.42 on day one and increased to 84.12 on day six (p=-0.389); and the average lymphocyte count was 19.37 on day one and decreased to 8.77 on day six (p=0.234) [29]. The average LCR increased from 1.64 on day one to 6.91 on day six (p=0.009), and the mean NLR increased from 11.79 on day one to 11.94 on day six (p=0.285) [29]. Although there were changes, the only statistically significant differences were in the average decreased CRP (p=0.005) and the increased LCR (p=0.009) [29]. Although these two differences prove the beneficial anti-inflammatory effect of aPRP in these patients, the other lab values neither prove nor disprove this, as they are statistically insignificant [29].

From 2009 to 2014 Alamdari et al. conducted a randomized controlled trial to evaluate the efficacy of pleurodesis with PRP and fibrin glue for the treatment of chylothorax post-esophagectomy [30]. A total of 52 patients diagnosed with esophageal cancer were included in this trial and were randomly assigned into two groups, with 26 patients in each group: one group underwent thoracic duct ligation via surgery and one group underwent pleurodesis with platelet-rich fibrin glue (PRFG) from PRP via chest tube [30]. Chylothorax refers to an accumulation of lymphomatous fluid within the lung pleura, commonly caused by damage in the thoracic duct [31,32]. Pleurodesis is a procedure to obliterate the pleural space between the visceral and parietal pleura of the lung [31,32]. PRFG was created by first centrifuging the blood to make PRP, as previously discussed. However, a portion was then centrifuged again to separate platelets from plasma, and this plasma combined with PRP constituted the final PRFG [30]. While all 26 PRFG pleurodesis patients were successfully treated, 20 of the 26 thoracic duct ligation patients had successful outcomes (p=0.009), although seven pleurodesis patients required an additional PRFG administration a week later [30]. The average hospital length of stay was 53.50 days in the thoracic duct group, compared to 36.04 days in the PRFG pleurodesis group (p<0.001) [30]. Additionally, the average ICU length of stay was 14.27 in the thoracic duct ligation group compared to 2.23 in the PRFG group (p=0.0001) [30]. The time to a successful outcome from the last intervention (surgery or PRFG) was also less in the PRFG group (3.90 days), compared to the thoracic duct ligation group (4.77 days), which was found to be statistically significant (p=0.004) [30]. Five patients in total died, four from the thoracic duct group and one from the PRFG group; however, this outcome was not statistically significant (p=0.1621) [30]. Statistical analysis was performed using the student’s t-test, Chi-square test, and Fisher's exact test, with p<0.05 considered statistically significant [30]. The results that showed statistically significant increased success rates and decreased hospital and ICU stay lengths suggest that pleurodesis with PRP and fibrin glue should be considered in all postoperative chylothorax patients [30].

In the only lab study reviewed here, Beitia et al. evaluated if nebulized PRP had a more beneficial effect on lung fibroblast growth compared to non-nebulized PRP [33]. Four healthy human donors were used for PRP collection. After the PRP was obtained, a calcium activator was added [33]. This article refers to the final solution as platelet lysate (PL), although there was no difference with activated PRP (aPRP) [33]. The PRP-PL was applied to lung fibroblast tissue contained in a fibroblast growth medium, via nebulization and non-nebulization (standard) [33]. A serum-free medium was used as a control. The researchers measured the growth rates of each for 96 hours, with data points collected at 24, 48, 72, and 96 hours [33]. Statistical analysis was performed using an unpaired t-test, with p-values under 0.05 identified as statistically significant [33]. Although the results indicate that both nebulized and non-nebulized PRP induced statistically significant fibroblast growth rates compared to controls, the study also showed that non-nebulized PRP has statistically higher rates than nebulized [33].

Between 2007 and 2012, Serraino et al. conducted a non-randomized controlled trial to evaluate the efficacy of PRP in preventing sternal wound infections in cardiac surgery patients [34]. After undergoing successful cardiac surgery, 671 patients, acting as controls, received standard closure of the chest, while 422 patients had the same closure but with PRP added to the edges of the sternum [34]. All patients had standard wound dressing changes daily for one month and received antibiotics prophylactically [34]. In the PRP group, two patients (0.5%) suffered from superficial sternal wound infection (SSWI), while the control group had 19 patients (2.8%) who experienced SSWI (p=0.006) [34]. In the PRP group, only one patient (0.2%) developed a deep sternal wound infection (DSWI), while the control group had 10 patients (1.5%) who experienced DSWI (p=0.043) [34]. Statistical analysis was performed using the unpaired t-test, with clinical significance determined by p-values <0.05 [34]. The control group had more incidents of deep and superficial sternal wound infections when compared to the PRP group [34].

Lastly, from 2009 to 2014, Huang et al. conducted a randomized controlled trial among patients with osteoarthritis of the knee to assess intra-articular PRP’s treatment capacity [35]. A total of 366 patients were randomly divided into two groups: 310 received PRP while 56 got the placebo (normal saline solution) [35]. All patients received 10 mL of either PRP or placebo via intra-articular injection with ultrasound guidance once a week, for a total of four weeks [35]. The following inflammatory plasma marker levels were evaluated: interleukin-17A (IL-17A), interleukin-1β (IL-1β), tumor necrosis factor-α (TNF-α), receptor activator of nuclear factor-κB ligand (RANKL), interleukin-6 (IL-6), and interferon-γ (IFN-γ) [35]. Additionally, the following plasma pro-angiogenic marker levels were assessed: hepatocyte growth factor (HGF), intercellular adhesion molecule-1 (ICAM-1), osteopontin (OPN), platelet-derived endothelial cell growth factor (PD-ECGF), VEGF, PDGF, insulin-like growth factor-1 (IGF-1), and TGF-β [35]. All lab values were taken at baseline (before injection) and again at eight weeks (four weeks post-injection) [35]. Additionally, MRI was performed at eight weeks to assess the degree of joint inflammation, synovial degeneration, and neovascularization [35]. At the eight-week assessment, all inflammatory marker levels were significantly lower in the PRP group compared to the placebo group (IFN-γ and IL-17A showed p-values <0.001; all other inflammatory markers had p-values <0.01) [35]. Conversely, at week eight, all pro-angiogenic marker levels were significantly greater in the PRP group compared to the placebo group (ICAM-1, OPN, and PDGF had p-values <0.01; all other pro-angiogenic markers had p-values <0.001) [35]. Joint inflammation, synovial degeneration, and percentage of the damaged area were significantly downregulated in the PRP group compared to the placebo on MRI (p<0.001), but neovascularization was significantly greater in the PRP group compared to the placebo (p<0.001) [35].

Discussion

The data from the 15 research articles that were used to test the hypothesis of this paper will be summarized and further discussed in this section. While four of the presented articles explore the relationship between PRP therapy and lung function improvement, which could decrease symptom severity and exacerbation, the remaining 11 studies expanded on the association between PRP treatment and anti-inflammatory qualities, which help accelerate the healing and regeneration of lung tissue. The following paragraphs will explore these connections further and describe the limitations of these studies. Table 1 summarizes all primary articles used in this study. It includes a summary of all published materials, except for articles that were used for background information in the Introduction section.

**Table 1:** Summary of all primary studies reviewed in this paper PRFG: platelet-rich fibrin glue; PRP-PL: platelet-rich plasma - platelet lysate; COPD: chronic obstructive pulmonary disease; PRP: platelet-rich plasma; RAO: recurrent airway obstruction; IAD: inflammatory airway disease; NL: normal lung; BAL: bronchoalveolar lavage; COVID-19: coronavirus disease 2019; ARDS: acute respiratory distress syndrome; aPRP: activated platelet-rich plasma; FEV1%: percentage of the forced expiratory ventilation within the first second

First author	Publication date	Study design	Study population	Therapy or exposure
Alamdari et al. [30]	April 2018	Randomized controlled trial	52 patients with chylothorax	Group 1: thoracic duct ligation (surgery); Group 2: pleurodesis with PRFG
Beitia et al. [33]	February 2021	Preclinical lab study	4 healthy adult donors	PRP-PL nebulized and non-nebulized (standard) on lung fibroblast cultures
Coleman and Rubio [21]	March 2018	Case series	568 COPD patients	Group 1: single PRP; Group 2: double PRP; Group 3: bone marrow and PRP; Group 4: booster
Dzyekanski et al. [28]	December 2012	Animal trial	10 racehorses divided into 3 groups: 1: RAO, 2: IAD, 3: NL	PRP was given intrabronchially and BAL fluid was assessed
Gómez-Caro et al. [26]	December 2011	Animal trial	15 adult Yorkshire pigs	Group 1: sham; Group 2: non-PRP; Group 3: PRP
Huang et al. [35]	January 2018	Randomized controlled trial	366 patients with osteoarthritis of the knee	Group 1: PRP injections; Group 2: placebo (normal saline) injections
Karina et al. [22]	July 2021	Case series	7 COVID-19 patients with ARDS (3 severe, 4 critical)	aPRP infusion on 1st, 3rd, and 5th day
Karina et al. [29]	July 2021	Phase I/II clinical trial	10 severely ill COVID-19 patients	aPRP infusion on 1st, 3rd, and 5th day in ICU
Maher et al. [27]	July 2020	Animal trial	70 Albino rats with amiodarone-induced lung fibrosis	Group 1: control; Group 2: amiodarone and phosphate buffer saline; Group 3: amiodarone and PRP
Mammoto et al. [24]	January 2016	Animal trial	12 mice with left pneumonectomy	6 mice were given PRP; 6 mice acted as controls
Pires et al. [23]	June 2021	Animal trial	37 racehorses with exercise-induced pulmonary hemorrhage	Group 1: placebo; Group 2: PRP
Rubio [19]	June 2021	Cohort study	419 COPD patients	PRP infusion
Rubio [20]	January 2021	Cohort study	281 COPD patients: Group A: FEV1% reassessed at 3 months; Group B: FEV1% reassessed at 12 months	PRP infusion
Salama et al. [25]	July 2019	Randomized controlled trial	40 adults with smoke inhalation injury: Group A: study; Group B: control	Group A: nebulized PRP therapy plus a normal regimen; Group B: only normal treatment regimen
Serraino et al. [34]	May 2013	Randomized controlled trial	1,093 post-cardiac surgery patients	Group 1: Control Group 2: PRP at the wound

Two cohort studies by Rubio were included in this paper. In her first article, results showed an improvement in the self-reported CCQ scores after PRP treatment, compared to their baseline [19]. Since the CCQ measures COPD symptom severity and exacerbations, mental health, and functional health state, the improved scores correlate with a better outcome in terms of respiratory symptom control and an improvement in the quality of life of patients [19]. Additionally, 150 patients out of the original 419 had their FEV1/FVC re-evaluated at three months, which also showed an improvement in their scores [19]. Although this cohort had a respectable sample size, the limitations of this study include the lack of a control group, which the author stated was due to ethical grounds in not wanting to not treat COPD patients [19]. Another limitation of this study was the limited number of patients whose FEV1/FVC was remeasured at three months [19]. However, the results of this study highlight the beneficial effect that PRP therapy has on lung function in COPD patients, supporting the hypothesis of this paper, although with low evidence.

The second article by Rubio evaluated the change in FEV1/FVC scores in 281 COPD patients after PRP therapy [20]. This study divided the patients into two groups: Group A (150 patients) had their FEV1/FVC remeasured at three months, and Group B (131 patients) had theirs retaken at 12 months [20]. The results showed an improvement in both groups’ FEV1 scores after treatment compared to their baseline scores [20]. These results also show the benefit that PRP has on COPD patients’ lung function through their improved scores; however, there are limitations to this study [20]. Apart from the limitation associated with the small sample size, this study also did not have a control group for similar reasons to the author’s previous study. While the findings in this cohort do support the hypothesis of this paper, this article also had a low level of evidence.

In the case series by Coleman and Rubio, PRP usage in COPD patients was also shown to be beneficial based on self-reported decreases in symptom severity, and improvement of mental and functional health [21]. Group 2 (double PRP treatment) showed the highest rates of improvement in symptoms and mental health than the other three groups, but Group 3 (bone marrow and PRP therapy) displayed the best improvement in functional health [21]. While this study included many subjects, it was also hampered by the lack of a control group, and the results should be considered with caution [21]. This study also supports our hypothesis.

In the case series by Karina et al., seven patients were given adjunctive-activated PRP treatment for COVID-19-induced ARDS [22]. Although two of the three tested measurements were shown to be statistically insignificant (IL-1B levels and lung injury scores) before and after treatment, the overall increase in PaO_2_/FiO_2_ of the seven patients signifies that the PRP treatment improved their lung function [22]. The average increase of 73.64 mmHg in their PaO_2_/FiO_2_ showed improved lung function, supporting this paper’s hypothesis [22]. However, this series also lacked a control group, and the results should be viewed cautiously.

In the randomized controlled trial conducted by Salama et al. involving 40 smoke inhalation patients, results showed that PRP had a positive correlation with lower intubation day lengths, lower hospital stay lengths, lower mortality rates, and an increased PaO_2_/FiO_2_ when compared to the control group [25]. The PRP group also showed evidence of decreased pulmonary edema, decreased mucus formation, and lower inflammation of the airway [25]. This study suggests that PRP use in smoke inhalation patients can cause anti-inflammatory effects and overall better tissue regeneration, which supports the hypothesis of this paper [25].

We examined five studies involving animal trials in this review, and they showed positive tissue regeneration and anti-inflammatory effects of PRP treatment. Although the tested animal populations (two studies on mice, one on pigs, and two on horses) were different, each study showed accelerated pulmonary tissue healing.

In the randomized controlled animal trial in racehorses by Pires et al., the results showed that PRP decreased the level of intensity of bleeding and inflammation [23]. Although the exact mechanism is still under investigation, it was inferred that the platelet’s effect on releasing growth factors, which are involved in inflammation, clotting, cell adhesion, and proliferation, played a major role in controlling the bleeds [23]. Evidence showed that exercise-induced pulmonary hemorrhage leads to extensive lung vasculature remodeling and angiogenesis, and the high concentration of platelets in PRP enabled the control of the deposition, causing a more favorable remodeling with less inflammation [23]. Although the inability to measure PRP and growth factors in these subjects is a limitation [23], this study does suggest a positive remodeling of lung tissue with decreased inflammation, which supports the hypothesis of this paper.

In the study by Mammoto et al., the PRP-treated mice showed significant results in comparison to the control group [24]. The results showed an overall acceleration of lung healing based on an increased lung-to-body weight increase by 14%, increased lung compliance by 30%, thickened alveoli septa, decreased alveolar space size, increased number of alveoli, and increased blood vessel density [24]. This data imply that PRP usage improved overall lung regeneration in unilateral pneumonectomy mice [24]. Even though this article tested mice, its findings could be applied to humans with lung disease as well, supporting the tested hypothesis of this review [24].

In the other animal trial on mice, Maher et al. used PRP in amiodarone-induced pulmonary fibrosis [27]. In the study, both groups treated with amiodarone suffered from thrombocytopenia and hemolytic anemia, which were induced by the amiodarone injections [27]. Although these groups had hematologic issues, the mice showed improvement after one week of PRP treatment, compared to the non-PRP-treated mice [27]. Additionally, the PRP-treated mice had an acceleration of lung tissue healing when compared to the non-PRP group [27]. Their results indicate that the PRP treatment accelerated the regeneration of the lungs, which caused the normalization of lung tissue, thereby supporting the hypothesis of this paper [27]. 

In the controlled animal study by Gómez-Caro et al., researchers applied PRP therapy for surgically induced anastomosis in adult pigs [26]. Their results indicated that the PRP itself had higher platelet-derived growth factors (TGF-β, VEGF, and EGF) compared to whole blood analysis [26]. These growth factors have been proven to accelerate tissue repair and healing. Additionally, their results showed higher blood flow rates across the PRP-applied anastomoses, compared to the non-PRP and controlled ones [26]. Although pathology reported higher vessel density and epithelial thickness in the PRP group, this was ultimately deemed statistically insignificant (p-value greater than 0.05) [26]. The higher growth factor levels in the PRP and the increased blood flow rates indicate a faster tissue repair in these pigs, which ultimately supports the hypothesis of this paper that PRP induces an acceleration of pulmonary tissue repair [26].

In the final animal trial presented in this paper, Dzyekanski et al. examined the use of PRP therapy in racehorses that had recurrent coughs [28]. The results indicated that the horses with inflammatory airway disease had significant benefits from PRP therapy [28]. They showed anti-inflammatory effects via a reduction in relative neutrophil count and tracheal mucus grade of their BAL sample, as well as a clinically significant improvement in their cough [28]. However, the groups with recurrent airway obstruction and the undiagnosed (normal lung) showed no significant effects with the PRP treatment [28]. This study was limited by the lack of a control group and the use of animals instead of humans, and hence these results should be reviewed carefully [28]. The results of Dzyekanski et al. do support this paper’s hypothesis, but with weak evidence.

In the phase-I/II clinical trial performed by Karina et al., the researchers tested the safety and efficacy of PRP infusions in 10 severely ill COVID-19 patients suffering from ARDS [29]. Although their results indicated that only two out of the five tested lab measurements were proven to be statistically significant [29], the study did show a reduction of CRP levels by an average of 8.36 and an average increase of the lymphocyte-to-CRP ratio to 5.27 after three treatments of PRP infusions [29]. Since CRP is a useful marker of inflammation, its reduction provides an important indication of anti-inflammation [29]. However, the other three tested measurements (average neutrophil count, lymphocyte count, and neutrophil to lymphocyte ratio) were statistically insignificant, and could not be used [29]. Although the results indicate the anti-inflammatory properties of PRP therapy in COVID-19 patients, this study lacked a control group and should be viewed accordingly [29]. However, this article supports the hypothesis of this paper, indirectly, via the anti-inflammatory effects provided by PRP.

The randomized clinical trial by Alamdari et al. involving post-esophagectomy chylothorax patients with PRFG showed a massive success for PRFG pleurodesis when compared to thoracic duct ligation surgery [30]. All 26 patients in the PRFG pleurodesis group were successfully treated, had a lower average hospital and ICU stay length, and had a quicker time to success after intervention [30]. The researchers’ rationale for administering PRFG was PRP’s well-evidenced wound-healing capabilities [30]. These properties of PRP are made possible by the release of many growth factors and cytokines, which play a vital role in chemotaxis, cell proliferation, and differentiation [30]. Through these capabilities, PRP accelerates the framework required in the process of establishing successful pleurodesis [30]. Though this study does not directly support the hypothesis of this paper, it underlines the effect that PRP has on cell proliferation and differentiation through its released growth factors.

The human clinical trial conducted by Serraino et al. evaluated the use of PRP in preventing sternal wound infection in post-cardiac surgery patients [34]. The results of this study show that the patients who were given PRP at the wound site had significantly lower rates of superficial and deep sternal wound infections, compared to the control group [34]. Although this study was a controlled human trial, it was non-randomized, and hence the results should be viewed accordingly with caution [34]. The authors of this article suggest that the rich concentration of platelets and the growth factors secreted caused an accelerated migration of inflammatory cells, leading to improved angiogenesis and overall healing [34]. This mechanism during the inflammatory stage of healing indirectly supports the hypothesis of this paper that PRP can regenerate lung tissue.

The clinical trial discussed by Huang et al. highlights the anti-inflammatory properties of PRP, which were demonstrated by the reduction in inflammatory marker levels, as well as the reduction of the degree of joint inflammation seen on MRI when compared to the placebo group [35]. Additionally, PRP induces angiogenesis and regeneration through the growth factors secreted by the high concentration of platelets, which was shown by the increased pro-angiogenic marker levels and the increased neovascularization on MRI compared to the placebo [35]. MRI also revealed a reduction of synovial degeneration, and the extent of damaged tissue was lower compared to the placebo [35]. Although this article does not discuss the effect PRP has on lung tissue, this trial was double-blinded with a high level of evidence and can better illustrate the effects of PRP [35]. This human clinical trial indirectly supports the hypothesis of this paper by highlighting the anti-inflammatory and tissue-regenerating effects that PRP has on the body.

The final article that we reviewed in this paper was a lab study by Beitia et al., which tested the effects of nebulized PRP over standard PRP on lung fibroblast growth [33]. Although the standard aPRP had higher growth activity than the nebulized aPRP, both nebulized and non-nebulized variants had significantly more growth than the serum-free control [33]. These results indicate that aPRP causes an acceleration of fibroblast growth rates, and because fibroblast cells can help induce the differentiation and growth of other pulmonary cells, particularly alveolar cells (type 1 and 2) and endothelial cells, the increase of fibroblasts is directly associated with an acceleration of the natural healing of the lungs [33]. This lab study supports the hypothesis of this paper that PRP therapy can be beneficial for chronic respiratory disease patients by helping with the regeneration of the lung tissue, which ultimately would slow the natural progression of the disease [33].

## Conclusions

The presented hypothesis of this paper - that PRP therapy can be beneficial for chronic respiratory disease patients to help regenerate the lung tissue, ultimately slowing the natural progression of the disease - has been proven through the evidence elicited from the 15 reviewed articles. Although not all chronic respiratory disease patients present in the same manner, the connecting link is that of damaged tissue of the lungs, causing issues with the functionality of the lungs. By adjunctively treating these patients with PRP, the high concentration of platelets and their growth factors can help induce an acceleration of healing and regeneration of pulmonary tissue. This, in turn, can slow the progression of the disease, which could lower the overall mortality rate in chronic respiratory disease patients.

We believe the findings of this paper pave the way for a potentially new area of treatment in chronic respiratory disease patients. While many animal trials show the benefits of PRP, more human studies are still needed. More studies should be conducted on this topic, specifically large, double-blinded, randomized human trials with controls to assess the efficacy and beneficial effects of PRP treatment on the lungs.
